# Smart and Functional Polymers

**DOI:** 10.3390/polym17243282

**Published:** 2025-12-11

**Authors:** Sixun Zheng

**Affiliations:** College of Chemistry and Chemical Engineering, Shanghai Jiao Tong University, Shanghai 200240, China; szheng@sjtu.edu.cn

In 2022, *Polymers* launched Section Smart and Functional Polymers as a platform to disseminate original ideas and concepts and to summarize the latest progress in the studies on smart and functional polymers. This section encourages original contributions on smart and functional polymers that mimic biological systems and have environmental and biological responses. In addition, it highly welcomes in-depth investigations into functional properties such as separation, electronic conductance, photo- and electro-luminescence, energy storage and conversion, data storage and so on. In the meantime, high-quality reviews on related topics are also accepted. Within the past two years, considerable attention from authors and readers has been attracted to this section, evidenced by many highly cited and accessed articles.

Inspired by functions of muscle organization, investigators have explored the fabrication of soft actuators with soft polymers. Such bio-simulating actuators can offer a wide variety of possible applications [1]. Shape memory alloys (SMAs) are a class of responsive materials which can memorize their original shapes from transient shapes. The combination of soft actuators with SMAs can significantly improve the usability of soft robots. Recently, Han et al. [2] developed a soft actuator that mimics the motion of an elephant’s trunk. In the actuator, a soft polymer was used for the construction of the body, whereas shape memory alloy springs were integrated into the body to provide a thermal response. This assembly can endow the soft actuators with flexible and diverse motion akin to an elephant’s trunk.

From the viewpoint of circular economy, the utilization of bio-mass-based materials is pivotal. As a waste byproduct of the avian egg processing industry, a great amount of eggshell membrane is available. Avian eggshell membranes represents a class of versatile biomaterials with chemical characteristics, structures and properties that can be further exploited for bone regeneration [3]. In addition, eggshell membrane particles have potential use as bio-ink for 3D printing of tailored implantable scaffolds. Recently, Álvarez-Lloret and Gómez-Morales et al. [4] summarized the extraction, biological and physical properties and calcium phosphate mineralization of eggshell membrane. This article was highly followed with interest. Flexible sensors are becoming the focus of a large number of investigations due to their vital application of intelligent detection and real-time data monitoring and recording. As an excellent biomass carbon-based material, flat silk cocoon is a good choice of flexible sensor. Recently, Cheng and Dai et al. [5] reported the preparation of a flexible piezoresistive sensor by encapsulating carbonized flat silk cocoons (CFSCs) with polydimethylsiloxane (PDMS). Notability, the sensor can effectively detect human motion with excellent durability. To fabricate resistive strain and pressure sensors, Wang et al. [6] integrated nanocomposites of PDMS with multi-walled carbon nanotubes (MWCNTs) into a smart glove for human motion/perception detection. To enhance the performance of capacitive sensors, Shen et al. [7] reported an approach of generating gradient structures with high-dielectric-constant media. By using laser-engraved acrylic molds and flexible copper-foil/polyimide-tape electrodes, a capacitive sensor was constructed with a gradient micro-cone architecture. Luo et al. [8] reported the preparation of hydrogel-based flexible sensors through the ultrafast polymerization of a hydrogel with a tannic acid–Fe^3+^ dynamic redox system. The hydrogel-based sensor had good adhesion performance. Furthermore, the sensors had a high-fidelity low detection limit, high sensitivity at small strains, and fast response and recovery.

In the fields of medicine and biotechnology, there has been a constant need to develop immunoassays, biosensors, and imaging techniques to provide detailed information for targeted disease diagnosis and treatment. Molecularly imprinted polymers (MIPs) can be taken as “antibody mimics”, which can be synthesized through the polymerization of functional monomers carrying “antigens” of interest or a derivative thereof [9]. In the past two years, the number of articles published on this topic has rapidly increased [10,11,12,13,14]. Such research involved various aspects, from chemical synthesis and theoretical computation to clinical applications of MIPs. Recently, a review by Wingren et al. [10,15] effectively covered the promising clinical applications of different MIPs recently developed for disease diagnosis and treatment.

Microcapsules are small capsules in which reactive liquid or solid materials are accommodated. Microcapsules have a variety of applications, such as self-healing materials [16] and smart protective coatings [17,18]. Smart coatings derived from functional polymers have garnered considerable interests due to their novel characteristics, such as self-restoration, self-cleaning, and self-healing behavior. Kothari and Iroh [16] recently reported a poly(urea formaldehyde)-based microcapsule, the self-healing behavior of which can be displayed at moderate and high temperatures. In addition, microcapsules are also applied in the field of smart protective coatings [17,18]. For instance, Yan et al. [18] reported a design of ultraviolet topcoat microcapsules, which were incorporated into the UV topcoat to obtain the paint films with robust mechanical strength and self-healing properties.

For food packaging, it is critical to develop sustainable materials to reduce the environmental impact of single-use packaging. In addition, it is also of interest to maintain the active characteristics of materials to extend the shelf life of products, enlarging their distribution period and reducing food losses along the distribution chain. Poly(hydroxyalkanoate)s (PHBs) are a class of linear polyesters, which are bio-based and biodegradable. Thanks to good thermomechanical and processing properties, they have been applied to obtain sustainable food packaging materials [19]. Recently, Carla Ivonne La Fuente Arias et al. [20] investigated the impact of phenolic compounds on the functional and structural properties of PHBs, and some new insights were obtained while the materials were used as packaging materials.

Compounding polymers with inorganics to obtain nanocomposites is an efficient approach to generating new materials with excellent thermomechanical and functional properties [21]. Polyhedral oligomeric silsesquioxanes (POSS) are a class of stereo molecules with cage-like Si-O frameworks, each silicon atom of which is bonded with one organic group (viz. R group) [22,23]. The incorporation of POSS into polymers to create such nanocomposites has provoked considerable interest. In the past years, this section has also published a number of articles that focused on smart and functional properties of POSS-containing nanocomposites. For instance, Raimondo et al. [24] demonstrated the combination of desired properties, such as electrical, flame-retardant and thermomechanical properties, through nanoscale incorporations of CNTs with POSS. For dental application, higher mechanical strengths and curing rates of materials are critical. Nanocomposites of methacrylic polymers with POSS can be used toward this end. Recently, Madhuranthakam et al. [25] investigated the interactions of mono- and multi-functional POSS with acrylic polymers through molecular dynamics simulations. It was found that the properties and curing behavior are highly associated with R groups and functionality of POSS macromers.

The development of environmentally and economically friendly polymeric materials has been the focus of a large number of investigations in recent years. Owing to the high demand for safety, there is a pursuit to utilize eco-friendly alternatives to synthesize next-generation fire retardants for building materials. Kolya and Kang [26] provided an overview of the current state of eco-friendly polymer nanocomposite coatings for fire retardancy, including the synthesis of nanocomposites, preparation, characterization, and the mechanisms underlying their fire-retardant properties. This review offers valuable insights for researchers and industry professionals in developing safer and sustainable fire-retardant systems. Cryogenic insulation is crucial for any cryogenic technology; rigid polyurethane foams with closed-cell structures have been successfully used as cryogenic insulation material for decades. However, it is still a challenge to develop environmentally friendly polyurethane forms towards sustainable cryogenic insulation. Cabulis et al. [27] summarized the progress in the studies of this field and provided good insight on utilizing bio-based polyols from renewable resources to synthesize polyurethanes.
